# Ex vivo microstructural investigations of oral lichen planus lesions by spectral domain optical coherence tomography

**DOI:** 10.1007/s10103-025-04493-w

**Published:** 2025-05-21

**Authors:** Alessio Gambino, Eugenio Giuseppe Martina, Francesca Spampinato, Giorgia El Haddad, Roberto Broccoletti, Luigi Chiusa, Paolo Giacomo Arduino

**Affiliations:** University of Turin, Turin, Italy

**Keywords:** Optical coherence tomography, Oral lichen planus, Histopathology, Optical biopsy

## Abstract

The aim of the work is to evaluate the Optical Coherence Tomography (OCT) capabilities in identifying the microstructural pattern of reticular Oral Lichen Planus (OLP) and compare it with the histopathological findings, to identify a common interpretation key and validate OCT as a diagnostic tool for this autoimmune inflammatory pathology. Eight patients were recruited (aged between 44 and 71 years). The anatomical sites chosen is the buccal mucosa, since it is typical of lichen lesions to be analysed. We took into consideration typical white signs of OLP: reticular and plaque lesions. Comparison between OCT scans and histological slides were carried out. Results show a strong correlation between OCT and histopathological evaluations. Hyperkeratosis occurs as a superficial hyperreflective zone. The lamina propria loses its hyper-reflective characteristic. This is probably due to the presence of the inflammatory infiltrate, which causes a decrease in signal strength. For the basement membrane, difficulties were encountered in interpreting it. This study shows that it is possible to identify clear differences between pathological tissue and healthy counterpart in OCT, both in epithelial and connective tissues. In addition, we observed a concordance in epithelial measurements between OCT image and histological image. These observations indicate promising potentials and need to be confirmed by further studies, in order to compare the results and arrive to an objective pattern of OLP, framing the possible role of OCT as a non-invasive diagnostic tool.

## Introduction

Oral Lichen Planus (OLP) is a chronic inflammatory mucocutaneous condition exclusive to the oral mucosa. It is an autoimmune disease characterized by a cell-mediated immunological dysfunction of T-lymphocytes against the cells of the basement layer [1].

OLP affects about 1–2% of the population, more frequently in females, with an F: M ratio of 1.5:1, and the age range is generally between 40 and 70 years [2]. OLP is also a potentially malignant disorder, with an estimated annual transformation rate to oral squamous cell carcinoma (OSCC) of around 0.20% [3]. Clinically, we distinguish two types of OLP: white OLP and red OLP. The white OLP is further divided, in increasing order of severity, into papular, reticular, and plaque forms, while the red OLP includes atrophic, erosive, and bullous lesions. Some authors consider the typical clinical features of OLP sufficient for a correct diagnosis, especially if classic skin lesions are present. The reticular variant is the more common of the two and presents with asymptomatic fine, white, linear, and lace-like lesions of the buccal mucosa and gingiva, referred to as Wickham striae [4].

To date, oral biopsy with histological examination is the gold standard for the diagnosis of OLP, and it is also necessary to exclude signs of oral dysplasia and malignancy [5]. Usually, an incisional biopsy must be performed on a white lesion: it is necessary to include both healthy and pathological tissue in the incision, and it is important to achieve adequate depth that includes the interface with the connective tissue [6].

The diagnostic criteria proposed in 2016 by the American Academy of Oral and Maxillofacial Pathology (AAOMP) for the diagnosis of OLP include:

1. Band-like or patchy, predominantly lymphocytic infiltrate in the lamina propria confined to the epithelium-lamina propria interface;
2. Basal cell liquefactive (hydropic) degeneration;
3. Lymphocytic exocytosis [7].

Early detection of malignant transformation of OLP during follow-up is difficult. For this reason, a confirmatory biopsy with histopathological examination is often necessary. This practice may appear more invasive in patients who present homogeneous or non-homogeneous lesions spread throughout the oral cavity, as is typical of OLP [8].

Therefore, some research has focused on finding non-invasive tools that can assist clinicians in the diagnosis of oral potentially malignant disorders (OPMDs) [9–10].

In recent years, Optical Coherence Tomography (OCT), a non-invasive optical technique for the ultrastructural imaging of malignant lesions or OPMDs, has become widespread with a good percentage of diagnostic sensitivity [11–12]. OCT allows to improve the management of OPMDs not only in diagnostic phase but also in the therapeutic stages (surgical or medical) [13–14].

**Fig. 1:**
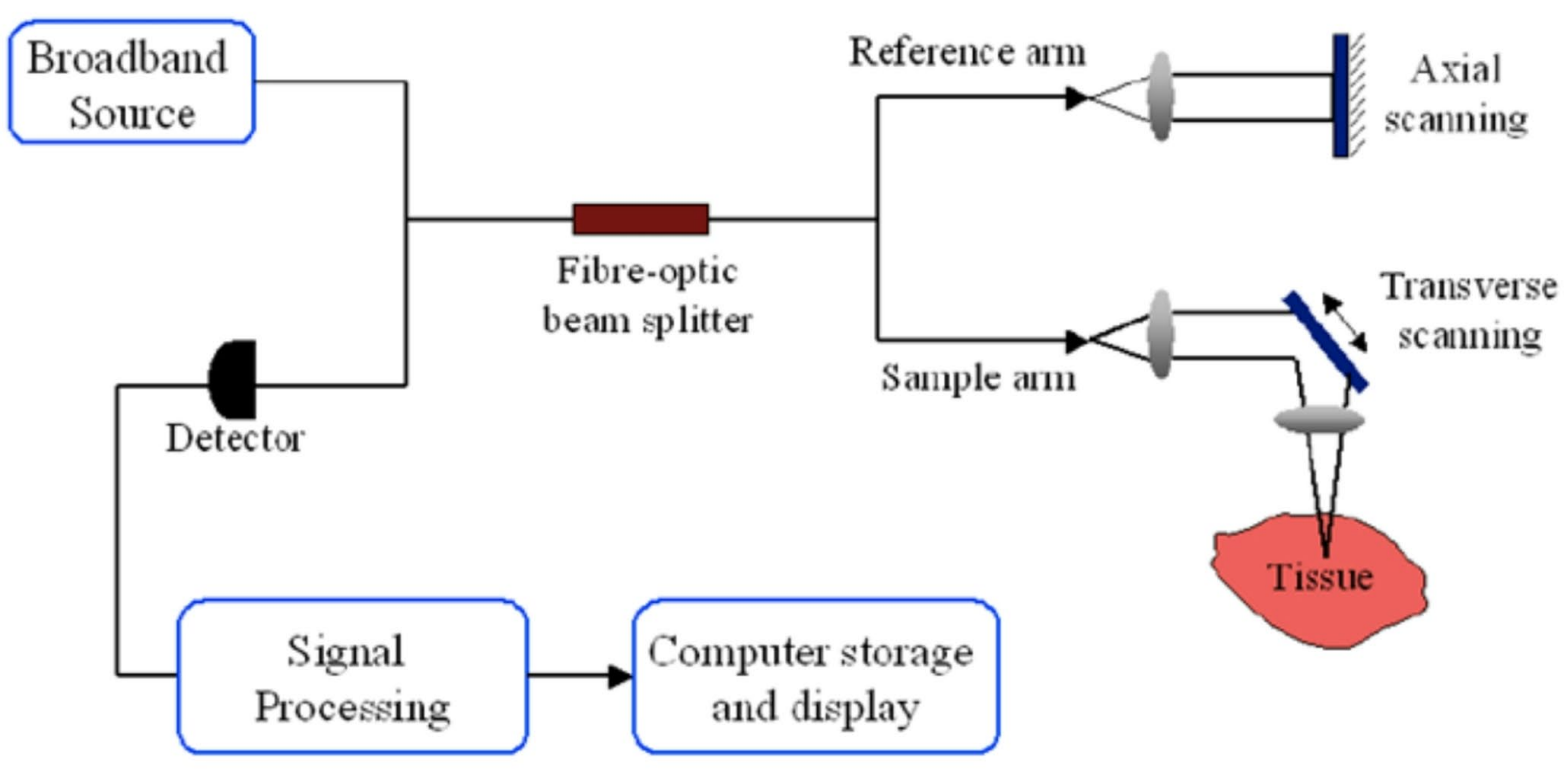
Example of a standard OCT system

OCT is a device similar to ultrasonography but uses a beam of laser radiation in the near-infrared frequencies (from 770 to 1300 nm) to measure the intensity of backscattering by the tissues (Fig. 1). OCT produces cross-sectional images of tissue, in 2D or 3D, with micrometer-scale resolution, thus providing “optical biopsies” that approach the resolution of histopathological images. OCT can be applied for in vivo and ex vivo evaluations, offering high-resolution images, generally between 1 and 20 μm of axial and transverse resolution, while the beam penetrates about 1–2 mm in thickness [15]. There are mainly two OCT methods: Time-Domain (TD-OCT) and Frequency-Domain (FD-OCT). According to the different acquisition modes, FD-OCT is divided into:

- Spectral Domain OCT (SD-OCT): The source used is broadband, and the resulting spectrum is measured by a spectrometer. This instrument breaks down the electromagnetic radiation into its components corresponding to various wavelengths, allowing for the simultaneous measurement of intensity by an array of sensors.
- Swept Source OCT (SS-OCT): The light source is a laser with a very narrow band and tunable frequency. The intensity data is recorded sequentially through a single sensor [16].

The diagnostic capacity of OCT in identifying various ex-vivo pathologies of the oral mucosa has been investigated in several studies [17]. The aim of this work is to evaluate the capabilities of OCT in identifying the microstructural pattern of reticular Oral Lichen Planus and to compare it with histopathological findings, in order to identify a common interpretative key to validate OCT as a non-invasive diagnostic tool for this autoimmune inflammatory pathology.

## Materials and methods

The study was carried out at the Department of Surgical Sciences – Oral Medicine Section – CIR Dental School, University of Turin, Italy. It was conducted in accordance with the principles of the Helsinki Declaration of 1975, as revised in 2000, and has been registered with ISRCTN (#17893224). Informed consent was obtained from all participants and/or their legal guardians. Ethical approval was granted by the Institutional Review Board of CIR Dental School, University of Turin. This is a qualitative research study that adheres to the EQUATOR guidelines for research reporting, using the Standards for Reporting Qualitative Research (SRQR) checklist for the collection and processing of instrumental and clinical data [18].

### Participants and sampling

A study group of 8 patients were recruited (3 males and 5 females, aged between 44 and 71 years) with oral lesions compatible with OLP was recruited in a period between May 2024 and July 2024.

The signs considered were reticular lesions/striae and white papules.

Exclusion criteria were:

- Oral lesions clinically compatible with OSCC.
- Oral lesions clinically compatible with Leukoplakia or other OPMDs.
- Patients smokers.
- Non homogeneous, ulcerative and/or erosive lesions.
- Oral lesions clinically compatible with erosive-atrophic OLP.
- Oral lichenoid-like lesions in patients affected by Graft-versus-host disease (GVHD).

**Fig. 2:**
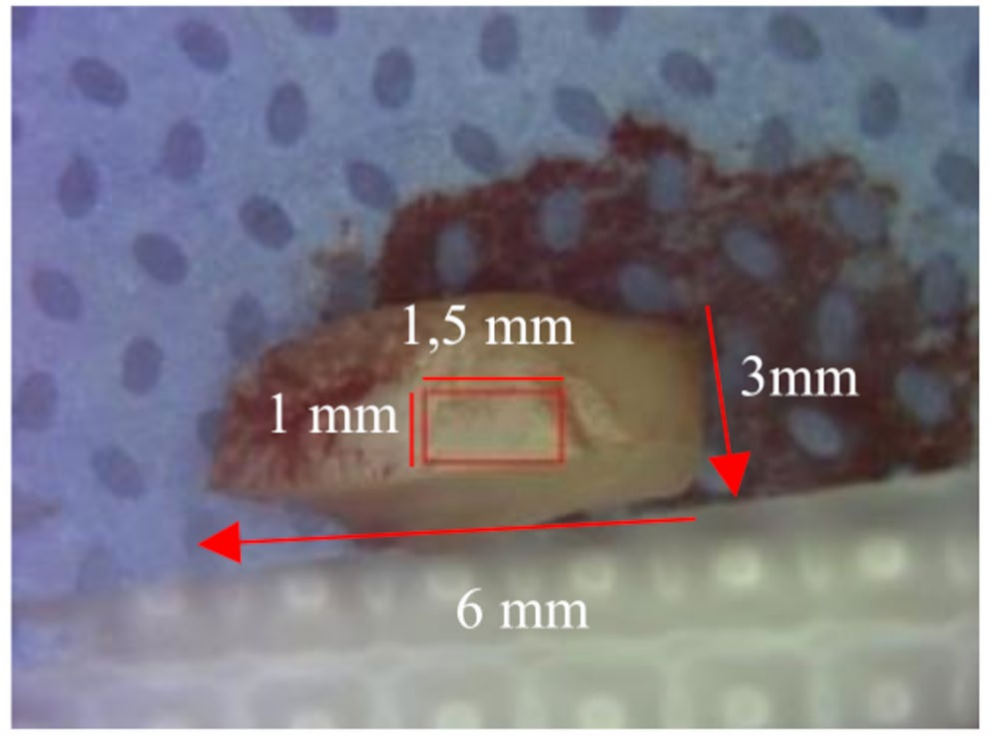
Biopsy sample after sectioning: the scanned part of the OCT system is highlighted within the red box

A biopsy was taken from the buccal mucosa as anatomical sites representative of OLP A punch biopsy of 6 × 6 mm diameter was performed for each patient. Subsequently, samples were sectioned keeping perpendicular to the to the central major axis such as to obtain a tissue loss of approximately 3 mm in width and 6 mm in heigh, in order to obtain a tissue loss of approximately 3 mm in width and 6 mm in height. In this way, the pathologist was able to identify and describe precisely the exact superficial section of the biopsy fragment scanned by OCT which corresponds to 1.5 × 1 mm of scanned tissue as shown in Fig. 2. The 3D scanning time was 10 s.

Control group was selected among patients aged similar study group, referred to our Department for excision of traumatic benign lesions of the buccal mucosa (i.e. irritational fibroepithelial polyp, or fibrous hyperplasia). A wider diameter of the surrounding healthy buccal mucosa was included in the original specimen, in order to be evaluated as healthy mucosa by the pathologist. A specific informed consentform was filled and signed by these patients, coupled with the usual informed consent required for biopsy. The sample was then subjected to histopathological analysis, carried out by the Pathological Unit - Città della Salute e della Scienza, Turin, Italy.

### SD-OCT system setup

Thorlabs’ OCT System Telesto 220 was used for our investigation.

**Fig. 3:**
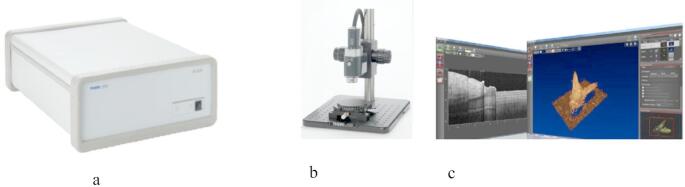
Telesto system OCT software

This system comprises three main components:

- *A spectral-domain (SD)* base unit (Fig. 3a);
- *A standard scanner (OCTG)* (Fig. 3b);
- *A PC*, in which the ThorImageOCT software is installed. (Fig. 3c).

**Fig. 4:**
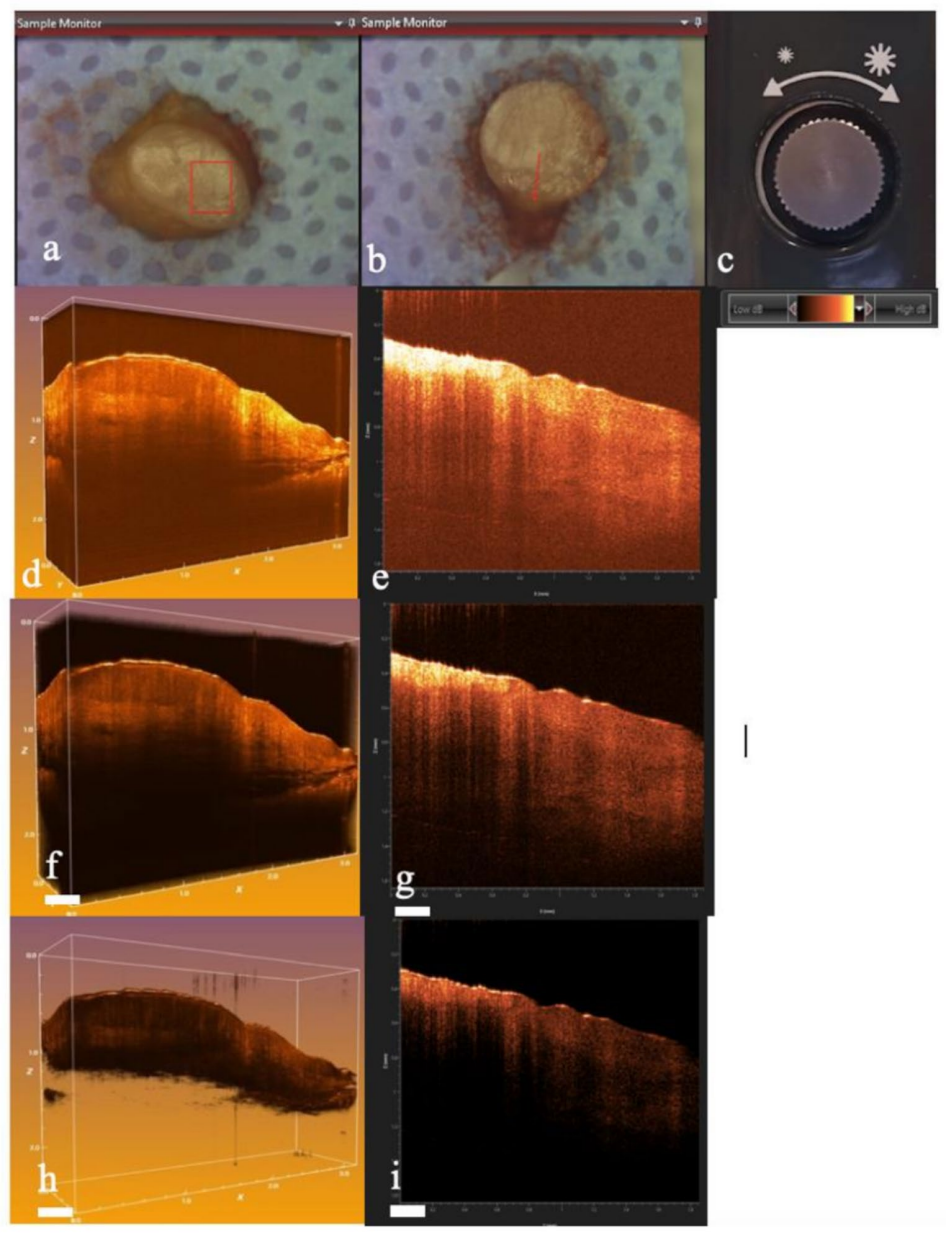
Selection for 3D (**a**) and 2D (**b**) scan modality with regulation knob (**c**); dynamic range between 20 and 60 dB in 3D (**d**) 2D (**e**); dynamic range between 30 and 70 dB in 3D (**f**) and 2D (**g**); dynamic range between 40 and 80 dB in 3D (**h**) and 2D (**i**)

The region of the biopsy specimen to be scanned visible as a red square area (if 3D) or a red line (if 2D) (Fig. 4a, b). The scan display modes can be adjusted to allow the “dynamic range”, expressed in decibels (dB), the brightness and the appropriate color to have a clear interpretable view of the image (Fig. 4c). The minimum value in dB that can be set is -10 dB, while the maximum is 110 dB. following parameters: 1300 nm wavelength, 10 mm x lateral resolution, and maximum image depth generally around 2 mm.

This system allows different modality of scanning:

- 2D (cross-sectional), where the reflective profiles are collected in the direction of depth, obtaining a longitudinal image along the x and z axis.
- 3D, capturing quadrangular volumes that allow transverse images to be displayed along the three x, y, z axes.

The best image quality for performing the analysis was set in a range between 30 and 70 dB.

### Evaluation criteria

The following analyses were carried out:

- Comparative analysis of healthy and OLP tissue in OCT, to identify the main ultrastructural alterations in the epithelium (EP), the lamina propria (LP) and the basement membrane (BM). Specifically, we considered the differences in thickness and reflectivity.
- Comparative analysis of healthy and OLP tissue in OCT and histological counterpart.

For each OCT image have been evaluated:

- Presence and thickness (mm) of the keratin layer.
- Presence and thickness (mm) of the epithelial layer.
- Reflective intensity (dB) of the epithelial layer.
- Presence and thickness (mm) of the lamina propria.
- Reflectance intensity (dB) of the lamina propria.
- Determinability of the basement membrane.

The images obtained from the OCT scans were compared with the corresponding images of histological preparations stained with hematoxylin-eosin view under a light microscope with 10x resolution.

In the Table 1 the interpretative criteria of the healthy tissue OCT images are summarized.

**Table 1:** Diagnostic-interpretative criteria of OCT scans

HYPER-REFLECTANCE	HYPO-REFLECTANCE	NON-REFLECTANCE
Keratin	Basement cells	Vessels/Liquids
Lamina propria	Epithelial layers	Epithelial tissue disorganization
Collagen fibers	Keratinocytes in maturation	No signal

### Data analysis

Statistical analysis was performed using Mann-Whitney test with Wilcoxon signed-ranked applied to analyze the single sample with two paired measures of non-parametric data. A 95% confidence interval and a 5% significance level (*p* < 0.05) were considered.

## Results

All histological evaluations confirmed the diagnosis of OLP.

In the OCT scans of OLP tissue it was noticed the loss of the characteristic hyper-reflectivity of the lamina propria, typical of a healthy oral mucosa, and an increase of the epithelial reflectance and thickness.

The mean of the measurements for the healthy epithelium is 0,24 mm, while for the OLP epithelium is 0,31 mm.

The lamina propria of OLP tissue turns out to be, on average, thicker than the healthy counterpart.

The mean of the measurements of the healthy lamina propria is 0,44 mm, while for OLP tissue it is 0,49 mm (Table 2).

Table 3 shows the reflectance values we found in healthy and in OLP epithelium and LP: in all 8 OCT samples a higher reflectance value in the OLP epithelium was found in most of the samples examined, the lamina propria appears to have a lower signal intensity in the OLP tissue than in the healthy one. in all samples, the signal intensity of the epithelium is higher than the lamina propria.

**Table 2:** Mesuraments (mm) of healthy and OLP tissue

Num. sample	H-EP (mm)	OLP-EP (mm)	H-LP(mm)	OLP-LP (mm)
Comparison 1	0,20	0,39	0,48	0,37
Comparison 2	0,25	0,37	0,39	0,47
Comparison 3	0,22	0,51	0,45	0,49
Comparison 4	0,27	0,22	0,42	0,31
Comparison 5	0,29	0,28	0,44	0,76
Comparison 6	0,24	0,28	0,40	0,49
Comparison 7	0,30	0,31	0,43	0,47
Comparison 8	0,20	0,36	0,52	0,55

H-EP: Healty Epithelium; OLP-EP: Oral Lichen Planus Epithelium; H-LP: Healthy Lamina Propria; OLP-LP: Oral Lichen Planus Lamina Propria

**Table 3:** Values of reflectance (dB) in healthy and OLP tissue

Num. sample	H-EP (dB)	OLP-EP (dB)	H-LP (dB)	OLP-LP (dB)
Comparison 1	52,0	61,2	59,4	50,7
Comparison 2	55,3	64,9	58,4	43,9
Comparison 3	55,9	62,1	66,2	45,8
Comparison 4	53,1	68,8	58,3	51,1
Comparison 5	47,6	48,4	55,2	43,5
Comparison 6	45,2	69,1	52,1	45,9
Comparison 7	49,9	67,3	62,7	51,3
Comparison 8	53,2	67,5	61,3	49,5

H-EP: healty epithelium; OLP-EP: Oral Lichen Planus epithelium; H-LP: healthy lamina propria; OLP-LP: Oral Lichen Planus lamina propria

Loss of reflectance in OLP-LP, in some cases, make it difficult to identify the basement membrane in OCT which was untraceable on 3 samples. On the other hand, the hyper-reflectivity of the epithelium facilitates it. Regarding the comparison between OCT and histological evaluation (Table 4), hyperkeratosis was detected on 3 OCT scans, while it is present in all histological samples.

The analysis of the epithelial layer shows a difference between histology slide and OCT scan measurements of less than 0.05 mm. The measurements of the lamina propria shows a difference between the two counterparts that is greater than 0,05 mm: the lamina propria measured in histological slides is thicker than the same structure measured in OCT.

**Fig. 5:**
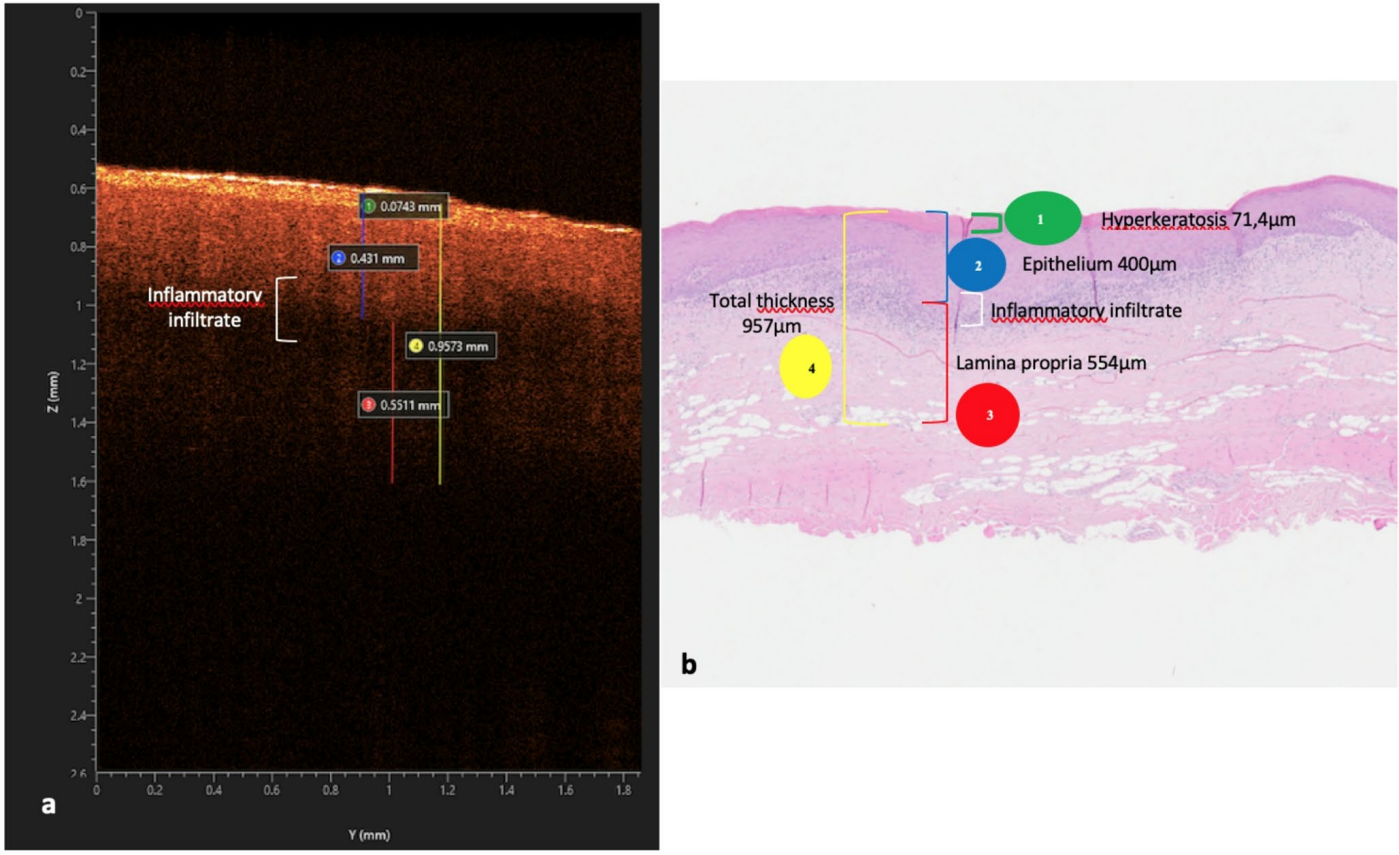
Representative image for measurements in OCT (**a**) and in traditional histology(**b**)

The average of the measurements of the lamina propria in OCT, in fact, is 0.49 mm, with a maximum value of 0.92 mm in a single bioptic sample. (Fig. 5)

**Table 4:** Comparison between OCT and traditional histology measurements of the OLP tissue

Sample	KL-OCT (mm)	KL-HY (mm)	EP-OCT (mm)	EP-HY (mm)	LP- OCT (mm)	LP-HY (mm)
Comparison 1		0,04	0,39	0,32	0,37	0,37
Comparison 2		0,02	0,37	0,32	0,47	0,51
Comparison 3	0,06	0,07	0,51	0,49	0,49	0,48
Comparison 4	0,05	0,07	0,22	0,29	0,31	0,62
Comparison 5		0,05	0,22	0,29	0,34	0,42
Comparison 6	0,21	0,20	0,28	0,33	0,76	0,81
Comparison 7	0,11	0,09	0,22	0,21	0,42	0,57
Comparison 8		0,03	0,28	0,33	0,49	0,49

KL-OCT: keratin layer in OCT; KL-HY: keratin layer histology; EP-OCT: epithelium in OCT; EP-HY: epithelium histology; LP-OCT: Lamina propria in OCT; LP-HY: lamina propria in OCT

**1** Hyperkeratosis (green line), **2** Epithelium (blu line), **3** Lamina Propria (red line), **4** Total thickness (yellow line).

The statistical investigation reveals a significant difference (*p* < 0.05) in thickness (mm) between healthy tissue (H-EP) and OLP (OLP-EP). No significant difference is observed in the LP section between healthy tissue (H-LP) and OLP (OLP-LP). Regarding the reflectance index expressed in dB, a statistically significant difference was detected between healthy tissue and OLP in both the EP and LP sections. This suggests that the OCT evaluation of healthy and OLP tissue is more pronounced in the EP section, and the difference in reflectance gradient may be valuable in distinguishing the pathological tissue. The comparison of measurements in the KL, EP, and LP sections between OCT and traditional histopathology shows a weakly significant difference only for the LP compartment (Table 5). Another observation pertains to the rounded shape of the biopsy sample, which may influence the way the light beam interacts with it, thereby affecting the quality of the scans.

**Table 5:** Statistical analysis using Mann-Whitney test with Wilcoxon signed-ranked

	H-EP (mm) OLP-EP (mm)	H-LP(mm) OLP-LP(mm)	H-EP (dB) OLP-EP (dB)	H-LP(dB) OLP-EP (dB)	KL-HY (mm) KL-OCT (mm)	EP-HY(mm) EP-OCT(mm)	LP-HY (mm) LP- OCT(mm)
*p* value < 0.05	*p* = 0.022	*p* = 0.240	*p* = 0.012	*p* = 0.015	*p* = 0.078	*p* = 0.572	*p* = 0.046

HP-EP: healty epithelium; OLP-EP: oral lichen planus epithelium; H-LP: healthy Lamina propria; OLP-LP: oral lichen planus Lamina propria KL-OCT: keratin layer in OCT; KL-HY: keratin layer histology; EP-OCT: epithelium in OCT; LP-OCT: Lamina propria in OCT; LP-HY: Lamina propria in OCT

## Discussion

The diagnostic ability of OCT in OLP lesions was evaluated in a previous study: an in-vivo comparative investigation involving 20 patients with atrophic-erosive OLP and 20 healthy subjects [19]. This investigation revealed distinct ultrastructural differences between cases and controls, showing a strong correlation between OCT and histopathological evaluations. A decrease in epithelial thickness, hyperparakeratosis, and loss of integrity of the basement membrane were observed. A hyper-reflective surface was identified with immediate hypo-reflectivity beneath, likely due to hyperparakeratosis and acanthosis of the epithelium, while the hypo-reflectivity at the level of the lamina propria is attributable to the presence of the inflammatory infiltrate. It has also been reported that the detectability of the basement membrane and the thickness of OLP are influenced by the inflammatory infiltrate and by apoptosis of the supra-basal and sub-basal cells, accompanied by capillary proliferation, which is typical of OLP [20–21]. The study by Gruda et al. describes the typical white OLP pattern, considering the OCT aspects and comparing its different tissue layers with those of histological slides, yielding results similar to those of our study, thus differentiating OLP from other OPMDs [22]. However, our study performed measurements of the various epithelial and subepithelial compartments to make the values more objective and comparable, as suggested by Prestin et al. [23]. Histopathology has remained the gold standard in the diagnosis of OLP, where the diagnosis is confirmed by biopsy through an invasive procedure followed by microscopic review. Furthermore, the samples represent only a very small part of the lesion, requiring clinicians to focus on the most significant areas, even in homogeneous lesions diffused in oral cavity [24]. The utility of the OCT is superior to other similar devices because allows an evaluation of the intraepithelial and sub-basal layers very close to traditional microscopy [25]. The choice to select homogeneous plaque and reticular lesions of OLP reflects the need to optically characterize lesions that may present with sometimes ambiguous clinical aspects, potentially indicating signs of malignant transformation [26]. The presence of parameters that identify dysplasia can be predictive of site-specific OLP lesion transformation and can aid in clinical evaluation, as well as inform the urgency of performing a biopsy or extending the sample [27]. The evaluation of the presence and/or absence of dysplasia is certainly the noblest action that OCT could provide to the clinician: for this reason, the construction of patterns that identify OLP without dysplasia is the first step to understand the patterns of OLP with dysplasia [28]. OCT has demonstrated high diagnostic performance with good agreement with histopathology regarding measurements of epithelial thickening, non-perceptible basement membrane, and architectural disorganization, making the measurement of the intra- and sub-epithelial compartments in OCT scans a significant diagnostic advancement [29]. OCT’s diagnostic accuracy to recognize OSCC has been highlighted by several studies [30]. A loss of normal characteristics of the oral mucosa layers compared to physiological images supporting the diagnostic process and the development of specific optical structural models applied to oral carcinogenesis in such a way as to identify dysplastic or malignant histological changes [31]. A few of studies have investigated the difference between OLP and other OPMD, including atrophic-erosive OLP and micro-invasive OSCC and providing a site-specific interpretation for differential diagnosis between these types of lesions [32]. Although the statistical analysis did not show any particular evidence in the comparison between traditional histology and OCT images, probably due to the limited number of samples, this ex-vivo study can be considered preliminary to a subsequent in-vivo study, especially for 3D applications, as it allows for more precise comparisons between healthy and non-healthy tissue in a manner similar to traditional histology [33–34]. This work also analyzed and quantified the reflectance values expressed in dB specifically for OLP. A few studies have shown that differences in the contrast of OCT images enable the characterization of OPMDs, which could complement the current criteria based on morphology alone in interpreting OCT images through the correlation between the differences in the reflectance gradient of epithelial cells and the corresponding ultrastructural layers. In particular, high-scatter or hyper-reflecting signals are associated with the flattening of nuclei and accumulation of cytoplasmic glycogen, while low-scatter or hypo-reflecting signals are more challenging to interpret [35–36]. For this reason, assigning a precise value to the reflectance parameter could make the interpretation of OCT scans more accurate and, in addition, could standardize the patterns of the different forms of oral mucosal pathologies [37]. Furthermore, in this work, we identified some limitations associated with this device. First, it is clear that an adequate learning curve is necessary for both the use of OCT and the interpretation of scans. We also observed that the scanning depth is limited to about 2 mm; however, considering that the band lymphocytic infiltrate is located close to the epithelium-stromal junction, it is reasonable to conclude that this does not preclude the use of OCT as a possible diagnostic tool for this pathology.

## Conclusion

This study confirms the reliability of OCT images in describing the ultrastructural patterns of OLP, distinguishing them from healthy tissues. However, OCT has limitations in image interpretation and inter-operator variability. Nevertheless, it is clear that its integration into clinical practice could enhance the accuracy of lesion assessments and improve patient outcomes by enabling physicians to select optimal biopsy sites and determine the degree of urgency during follow-up. The accuracy in describing the pattern of OLP without dysplasia, as shown in our case series, it is essential for a correct distinction from other OPMDs and especially from OLP with dysplasia.

Further ex-vivo and in-vivo studies will be needed to better understand the ultrastructural patterns of OPMDs, with precise measurements of the thicknesses of various intra-tissue compartments and greater accuracy in assessing the different degrees of intraepithelial and sub-epithelial reflectance. This approach will enable the device to compensate for the inability to accurately observe cellular abnormalities and to validate the diagnostic safety of this technology.

## Data Availability

No datasets were generated or analysed during the current study.
